# Open-Path Laser Absorption Sensor for Mobile Measurements of Atmospheric Ammonia

**DOI:** 10.3390/s23146498

**Published:** 2023-07-18

**Authors:** Soran Shadman, Thomas W. Miller, Azer P. Yalin

**Affiliations:** 1 Department of Mechanical Engineering, Colorado State University, Fort Collins, CO 80525, USA; 2 TCB Engineers, Surprise, AZ 85374, USA; tmiller@tcbengineers.com

**Keywords:** ammonia, open-path sensor, laser absorption sensor, wavelength modulation spectroscopy, quantum cascade laser

## Abstract

Anthropogenic emissions of ammonia to the atmosphere, particularly those from agricultural sources, can be damaging to the environment and human health and can drive a need for sensor technologies that can be used to detect and quantify the emissions. Mobile sensing approaches that can be deployed on ground-based or aerial vehicles can provide scalable solutions for high throughput measurements but require relatively compact and low-power sensor systems. This contribution presents an ammonia sensor based on wavelength modulation spectroscopy (WMS) integrated with a Herriott multi-pass cell and a quantum cascade laser (QCL) at 10.33 µm oriented to mobile use. An open-path configuration is used to mitigate sticky-gas effects and achieve high time-response. The final sensor package is relatively small (~20 L), lightweight (~3.5 kg), battery-powered (<30 W) and operates autonomously. Details of the WMS setup and analysis method are presented along with laboratory tests showing sensor accuracy (<~2%) and precision (~4 ppb in 1 s). Initial field deployments on both ground vehicles and a fixed-wing unmanned aerial vehicle (UAV) are also presented.

## 1. Introduction

Anthropogenic emissions of nitrogen (N) compounds represent a major air pollutant posing significant risks to human health and environment [1,2,3,4]. The most abundant nitrogen containing atmospheric species due to anthropogenic activity is ammonia (NH_3_) at ~55%, oxides of nitrogen (NO_X_) at ~40% and nitrous oxide (N_2_O) at ~5%. Ammonia thus represents a major nitrogen emission species from agricultural sites with contributions from both synthetic fertilizer and animal waste [5,6]. The process of ammonia emission from agricultural lands is based on ammonification: a series of metabolic activities performed by bacteria or fungi to convert organic nitrogen (e.g., from animal excrement) to ammonia. For example, over 50% of the nitrogen fed to cattle in feedlots and dairies is converted to ammonia in the air, and thus, ammonia concentrations in the areas nearby animal feeding operations are often between 500 and 1200 ppb, which is 100 to 200 times larger than background conditions [7]. Due to the potential adverse effects of ammonia on ecosystem and air-quality, there has been recent attention to best-management practices for reducing atmospheric ammonia [8], for example, stopping or postponing certain activities (such as laying down fertilizer or flowing irrigation water) when meteorological conditions are expected to transport ammonia to sensitive regions.

Issues related to ammonia emission from agricultural sources and associated environmental impacts are of acute concern in our region of northern Colorado (though it is important that other sources, such as industrial and vehicular emissions, also contribute to the total ammonia burden [9]). In this region, the highest emissions occur in the northeastern region of the state due to the large number of concentrated animal feeding operations (CAFOs) in this area. These CAFOs are also quite proximate (~150 km) to Rocky Mountain National Park (RMNP). Research has shown the RMNP ecosystem has been affected by nitrogen deposition to the park through both wet and dry deposition by which nitrogen is added to the park ecosystem (soils, plants, lakes and rivers) [10,11]. An example negative outcome of the surplus nitrogen is enhanced growth of nitrogen-limited plants, at the expense of other species, which then dominate the vegetation.

Detailed study of the ammonia emissions, for example, to yield concentration data to feed back-trajectory calculations or to make local source emission measurements, requires appropriate sensor technologies. Measurement of atmospheric ammonia concentrations and its regional variations have been relatively limited due to two important reasons: (1) Ambient concentrations are low and vary widely, and (2) ammonia is a sticky gas that introduces significant inlet challenges for typical closed-path instruments [12,13,14]. Herein, we give a non-exhaustive summary of some exemplar atmospheric ammonia gas sensors. We focus our attention on “point sensors”, as opposed to path-integrated sensors, as these are generally more amenable to mobile use for versatile study of different locations and sources. Sensors based on chemical ionization mass spectrometry (CIMS) can provide adequate sensitivity and time resolution (<1 ppb in few seconds) but tend to be relatively complex and large for field deployment [15,16]. In contrast, optical approaches can allow relatively compact instrumentation. Various techniques based on near-infrared and mid-infrared absorption have been developed including direct absorption spectroscopy [17], photoacoustic spectroscopy [18], integrated cavity output spectroscopy [19], differential absorption spectroscopy [20], cavity ring-down spectroscopy [21,22,23] and wavelength modulation spectroscopy (WMS) with multi-pass cells [24].

Very few available or published sensors are suited for sensitive, mobile measurements of atmospheric ammonia, given such applications require that the sensor be quite sensitive (<~1 ppb) while also having fast temporal response (<~1 s), immune from chemical interferences and, particularly for use on unmanned aerial vehicles (UAVs), also being relatively compact, low mass and low power. An exception comes from the work of Miller et al. where WMS was used at ~9.0 μm to achieve favorable specifications (0.15 ppb detection limit at 10 Hz) in a sensor with favorable SWAP (size, weight and packaging) characteristics [24]. The sensor presented here builds off that work but operates in at wavelength of ~10.3 μm to access stronger absorptions. Our sensor development is also specifically oriented to achieving favorable SWAP in a robust design that can be used on mobile platforms. In particular, the advent of small UAVs in tandem with compact sensors provides great flexibility in accessing hard-to-reach or dangerous locations, achieving autonomous operation, and allowing scalable sampling campaigns [25,26]. The ammonia laser sensor developed in this study is relatively lightweight (<3.5 kg), low power (<40 W) with ppb level concentration sensitivity using open-path WMS.

The remainder of this paper is laid out as follows. Section 2 of this paper describes the setup as well as the theory and detailed methodology of the WMS approach. Section 3 describes laboratory results of sensor accuracy and precision. Section 3 also presents initial field deployments, both by ground vehicle and with a fixed-wing UAV (12-foot Telemaster). Finally, Section 4 presents Conclusions and Future Work.

## 2. Experimental Methods

### 2.1. Experimental Setup

**Figure 1:**
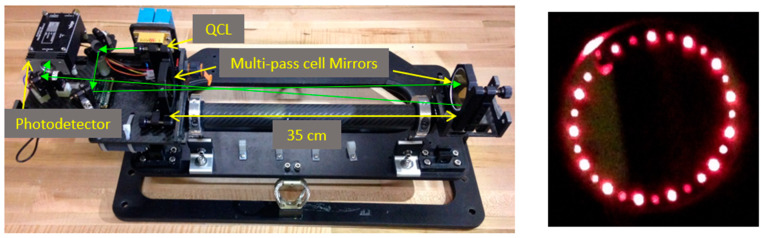
(**Left**): Open-path WMS based ammonia sensor. Green arrows show the approximate beam path (neglecting the multiple cell passes). (**Right**): Photograph of front mirror of multi-pass when illuminated with a red beam showing the 28 passes (input/output hole is top left + 27 beam spots).

Figure 1 shows the open-path ammonia sensor based on WMS with a quantum cascade laser (QCL) and a Herriot multi-pass cell. The light source is a single-mode, continuous-wave, thermoelectric-cooled, distributed feedback QCL (Corning) in HHL package with center wavelength at 10.33 μm, line width of a few MHz and output optical power of 30 mW. The laser temperature is stabilized by a thermoelectric temperature controller (Meerstetter, TEC-1091) and operated with a compact laser diode driver (Wavelength Electronics, FL500). As will be further discussed, the wavelength scan and WMS modulation are imposed via current control. The collimated laser beam is guided into a multi-pass cell through a hole in the first mirror. The multi-pass cell is a Herriot design [27] and serves to provide a longer effective path length to increase sensitivity. The multi-pass cell consists of two spherical mirrors (Thorlabs, CM508-200EH4-M02 and CM508-200-M02) with focal length of 20 cm, diameter of 50.8 mm, separated by 35 cm. The input/output hole is 4 mm in diameter and is 20 mm from the mirror center. By aligning the beam on a specific direction, it exits through the same hole (with a different slope) from the cavity after 28 roundtrips such that the effective path-length is 19.88 m. The right of Figure 1 illustrates the beam path in the cell by showing how it illuminates the front mirror (using a red laser beam). The cell output beam is finally focused by a germanium lens (focal length of 25 mm) onto the infrared-sensitive HgCdTe photodetector (VIGO PVM-2TE-10.6) active area (1 mm^2^). Efforts were made to have a lightweight and robust design for all components. Most mounts and physical structures are made from carbon-fiber to minimize thermal expansion. The overall size of the sensor is ~20 × 20 × 50 cm (~20 L).

All instrument control as well as the data acquisition (DAQ) system uses custom circuit boards coupled to a microcontroller (and the current/temperature controllers mentioned above). The microcontroller is a National Instruments sbRIO-9651 based on the Xilinx Zynq-7020 system on chip (SoC). These components are all housed within a 5 × 10 × 30 cm box. A custom LabVIEW program controls the DAQ system to simultaneously generate the analog input signal of the laser driver and to digitize the detector signal at 200 kHz sampling rate. Instead of a standalone hardware lock-in amplifier, a digital lock-in amplifier (also programmed within LabVIEW) is used to extract the 1*f* and 2*f* harmonics [28]. The same enclosure also houses sensors for temperature (OMEGA, RTD-806), pressure (Honeywell, PX2AM1XX001BAAAX) and geographical position (from Global Positioning System).

#### Sensor Mounting and Integration for Mobile Measurements

**Figure 2:**
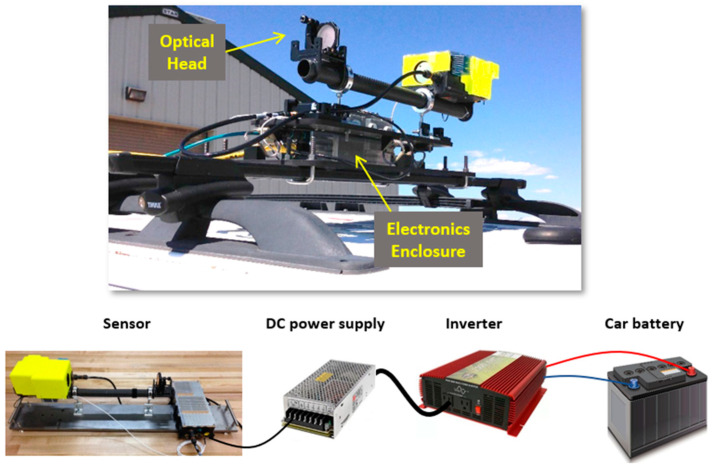
(**Top**): Photograph of sensor vehicle roof mount. (**Bottom**): Pictorial view of components used to power the sensor for vehicle deployments.

The sensor has been designed for both terrestrial (using a vehicle) and aerial measurements (using a UAS). Figure 2 shows the mounting and power scheme used for vehicle (truck) deployments. The sensor power draw is relatively low (<~30 W) such that it can be operated from a standard automotive battery while the vehicle is operating (without draining the battery). An inverter was first connected to the battery (~14–18 V) to provide fixed 110 V AC power. The inverter then powered a lab-style DC power supply (12 V) which in turn powered the sensor. (In other work, we have shown that we can also power direct from the battery with power-conditioning components). These power components are not outdoor-rated and are stored in the cab of the truck. The open-path sensor is deployed on the roof of the truck in order to sample the ambient air across the bam path of the open-path optical head. A custom aluminum anti-vibration mount was designed for mounting the sensor on the roof rack cross bars of the vehicle. The sensor and electronics enclosure are affixed to the mount with simple clamps and fasteners. The design was found to be quite robust to misalignment (on both paved and gravel roads) for the attempted test durations of 10 s of hours. The readings from the temperature sensor are used to correct for temperature dependences in the absorption spectrum as discussed in Section 2.2.

**Figure 3:**
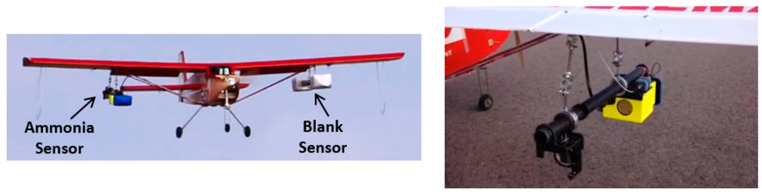
(**Left**): Sensor on flying UAV. (**Right**): Zoom-in (rear) view of ammonia sensor mounting.

Aerial deployment of air quality sensors can provide a means to reach hard-to-access locations (e.g., away from roadways) and/or a scalable manner of sampling many locations [25,26]. There can be advantages for use of both rotor-style UAVs (i.e., ease of use, no need for long runways) and fixed-wing UAVs (i.e., less flow perturbation due to the aircraft, in particular avoiding the problem of strong rotor downwash and associated turbulence, which can bias the sampling [29,30]). The present research has shown integration and test flights on a fixed-wing UAV (12-foot Senior Telemaster). Figure 3 shows integration of the ammonia sensor with the UAV. In this case, the optical head of the sensor is mounted under the wing of the UAV. The sensor is powered by a lithium ion battery (5000 mAh, ~14 V, ~0.5 kg), which can provide power for ~2.5 h, and both the battery and the electronics enclosure are housed within the central fuselage of the UAV (for better weight distribution). To balance the aircraft, a blank (dummy) sensor is used as a counterweight under the other wing. (As discussed below, in some cases, a methane sensor is used in place of the blank sensor.) As shown in Figure 2, an aerodynamic fairing (3-D printed blue piece) was placed on the front end of the sensor. We found that, with this mounting scheme, the sensors had minimal impact on the aerodynamics and ability to fly the UAV.

### 2.2. Principle of Operation and Analysis Method

The ammonia sensor is based on WMS detection via the strongest absorption line of ammonia in mid-infrared (MIR) spectral region (the Q branch of *v*_2_ bending band) [31,32]. At ambient pressure and temperature there is a very isolated (free from other species) absorption feature at 10.337 µm (967.35 cm^−1^) due to the rotational transition $\left(J=3,\;K=3\right)\to \left(J=3,\;K=3\right)$. The WMS technique has been described in detail previously [24,28,33,34,35], and here, we focus on elements specific to our implementation. WMS is fundamentally a laser absorption technique, where species concentrations are inferred through the Beer-Lambert Law, similar to direct absorption spectroscopy (DAS). One of the drawbacks of DAS is that its sensitivity is limited by low frequency noise in the signal, which originates mainly from the laser and detector noise. Also, in open-path configurations at atmospheric pressure, the absorption features are often not fully isolated such that determining the background (absorption-free baseline) signal can be challenging. These issues are addressed in WMS by modulating the laser wavelength (typically through laser current) at a relatively high frequency (typically ~10 kHz). This fast modulation is superimposed on the slower wavelength scan ramp (typically ~1–100 Hz) that serves to wavelength scan over the transitions absorption feature (as in regular DAS). The fast modulation, when combined with phase-sensitive (lock-in) detection, moves the measurement into a higher frequency regime where laser and detector noise (usually described by 1/f noise, or flicker noise) are greatly reduced thereby yielding higher signal-to-noise.

Another important feature of WMS is that it is a “derivative” technique, meaning that the 1*f* and 2*f* signals (i.e., the first and second harmonic signals found from the lock-in) closely resemble the first and second derivatives of the absorption spectrum. Measurements are therefore sensitive to spectral absorption shape or curvature rather than absolute absorption levels. This feature is particularly helpful for measurements in atmospheric pressure (or above) with non-isolated absorption features. For a robust measurement approach that is immune to laser power fluctuations (and atmospheric induced fluctuations, e.g., dust which can weaken the beam), appropriate signal normalization schemes can be used. Past research has shown that normalizing the 2*f* signal by the 1*f* signal is effective in this regard [28,33,34,36], and we follow such an approach.

We use a best-fit simulation approach, based on the 2*f*:1*f* spectrum, to infer ammonia concentration from measured signals [37]. A convenient feature (in addition to its strength) of the specific transition we employ is that it is very isolated, and though we include nearby absorption lines of other species (water and carbon dioxide), we find their effect to be completely negligible for our specific 2*f*:1*f* measurement of ammonia (for concentrations at ppb level and above). We can therefore simulate the 2*f*:1*f* spectrum for a fixed ammonia concentration and then seek the scaling factor that gives the best agreement between a measured spectrum and this reference spectrum to infer the measured concentration (equal to the scaling factor multiplied by the reference ammonia concentration). To account for atmospheric variation, which influences the signals partly and primarily due to line-broadening but also more weakly through the line intensity, reference spectra (at one NH_3_ concentration) are generated for 100 different combinations of ambient pressure and temperature (where we take 5 values of pressure spanning 0.81, 0.82 … 0.85 atm paired with 20 values of temperature spanning from 1, 2, …, 20 °C) based on typical conditions in our region. (We find from numeric simulation that the coarseness in our temperature and pressure discretization introduces at most 0.1 ppb of error in NH_3_). The simulated signal used is the ratio of the 2*f* amplitude to the 1*f* amplitude, denoted as *R*_2*f*_/*R*_1*f*_, where each amplitude is found from the in-phase (*X*) and (out-of-phase) quadrature (*Y*) components, i.e., $R_{nf}=\sqrt{{\left(X_{nf}\right)}^{2}+{\left(Y_{nf}\right)}^{2}}$ (where *n* = 1,2). The fast current modulation (10 kHz) also serves as the reference frequency input of the lock-in amplifier. The time-dependent QCL injection current is taken as the sum of a DC component (350 mA) plus a linear term (modulated at 100 Hz) for the main scan ramp plus the fast 10 kHz modulation:

(1) $$i\left[mA\right]=350-2000t+22\sin\left(2\pi \times 10^{4}t\right),\;\;\;\;0\leq t\leq 0.01\;s$$

**Figure 4:**
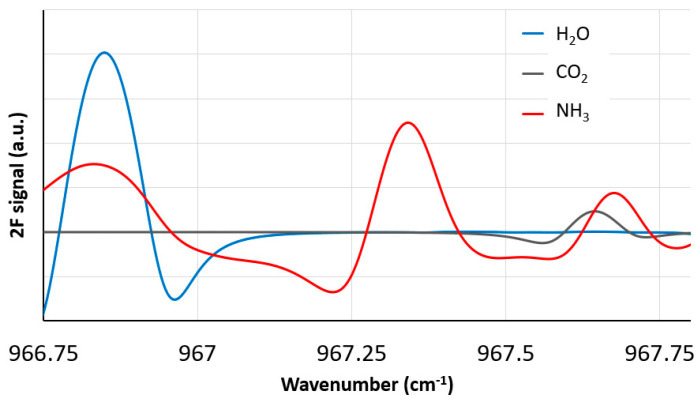

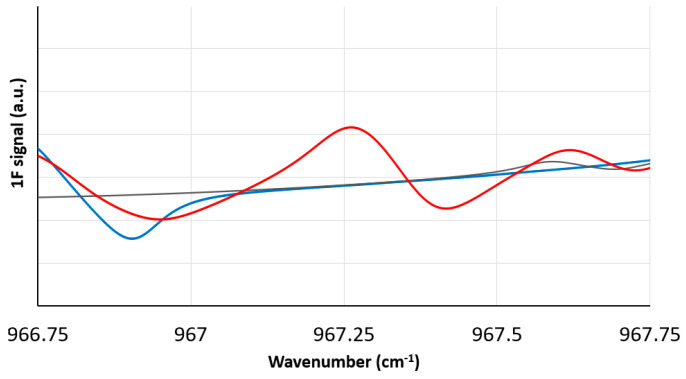
Simulated lock-in signals for 2*f* and 1*f* frequency amplitudes. See text for detail.

The modulation of injection current leads to simultaneous variation of laser wavelength and intensity. These functions were determined in separate experiments using an ammonia reference cell and etalon for establishing the frequency axis [37]. In particular, we find a phase difference of 1.08π between the laser frequency and the current modulation, which is similar to that obtained from other QCL characterizations. The injection current, measurement path length and spectroscopic parameters from HITRAN [32] allow calculation of the absorption and signal amplitude on the detector. The software lock-in (both for measurement and signal simulation) finds the in-phase and quadrature components by multiplying the (averaged) signal by sine and cosine waveforms at the reference frequency and phase (to create the *X* and *Y* components, respectively) and then passing the resulting waveforms through identical low-pass filters (1 kHz bandwidth) [36]. A given experimental measurement is based on averaging four cycles (40 ms) of detector signal. The time needed for a single measurement is found as 40 ms (4 cycles of the 100 Hz wavelength ramp) added to 160 ms of computation time (digital lock-in analysis and spectrum fitting) giving 200 ms (data rate of 5 Hz). Figure 4 shows an example of a simulated (unnormalized) in-phase 2*f* and 1*f* signal, i.e., *X*_2*f*_ and *X*_1*f*_, respectively, as function of laser wavelength in the vicinity of the ammonia absorption for the following conditions: P = 0.83 atm, T = 300 K, [NH_3_] = 100 ppb, [H_2_O] = 0.013 (50% relative humidity), and [CO_2_] = 390 ppm.

## 3. Results and Discussion

### 3.1. Laboratory Studies

#### 3.1.1. Example Measurement at Fixed Conditions

**Figure 5:**
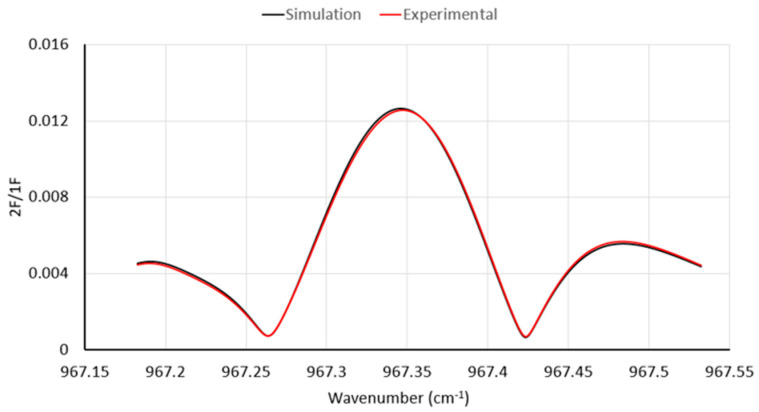
Comparison of simulated and experimental spectra for NH_3_ concentration of 150 ppb.

We first illustrate the workings of our WMS implementation by showing an example of a specific measurement simulation and result with gas delivered from a reference cylinder through a closed-cell cavity. Figure 5 shows a comparison of the WMS spectrum (2*f* amplitude normalized by 1*f*, i.e., *R*_2*f*_/*R*_1*f*_) for the case of NH_3_ concentration of 150 ppb (for P = 0.84 atm, T = 295 K, and L = 1988 cm for our multi-pass cell). The figure illustrates the scan range used in actual experimental measurements and shows an example of the data quality. One can see that there is excellent agreement in the peak amplitude and overall spectral shape, showing the validity of the assumed methods.

#### 3.1.2. Sensor Accuracy and Precision

**Figure 6:**
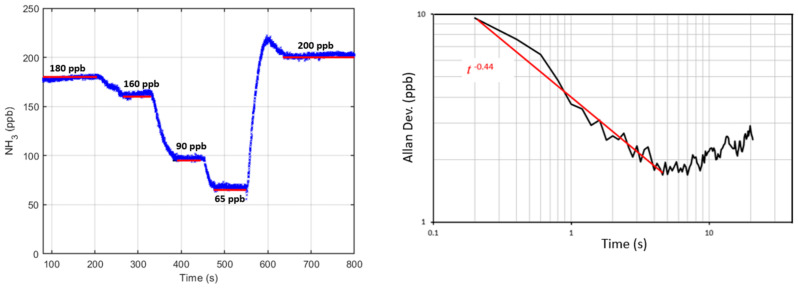
(**Left**): Comparison between measured and expected ammonia concentrations with closed-path configuration Red bars show the fix delivered concentrations. (**Right**): Allan variance analysis shows the sensitivity of ~4 ppb (at 1 s).

A closed-path configuration at atmospheric pressure was used to examine the precision and accuracy of the sensor. Zero-air (Airgas, 99.9996% pure) and ammonia gas (Airgas, 10 ppm ammonia diluted in nitrogen) from two reference cylinders were mixed and passed through the optical cavity. The left of Figure 6 shows a test characterizing sensor accuracy with very good agreement between the delivered and measured concentrations with data showing average discrepancy < 2% (and a plot of measured versus delivered concentration having slope of 0.985 and R^2^ = 0.9997). The right of Figure 6 shows an Allan variance study [38] yielding sensitivity of ~4 ppb (at 1 s), which is very adequate for many ammonia sensing applications. The Allan variance at early times is well fit with a *t*^−0.44^ dependence, close to what one expects in a shot-noise limited case (*t*^−0.5^).

### 3.2. Demonstrative Field Measurements

#### 3.2.1. Vehicle Based (Ground) Measurements

**Figure 7:**
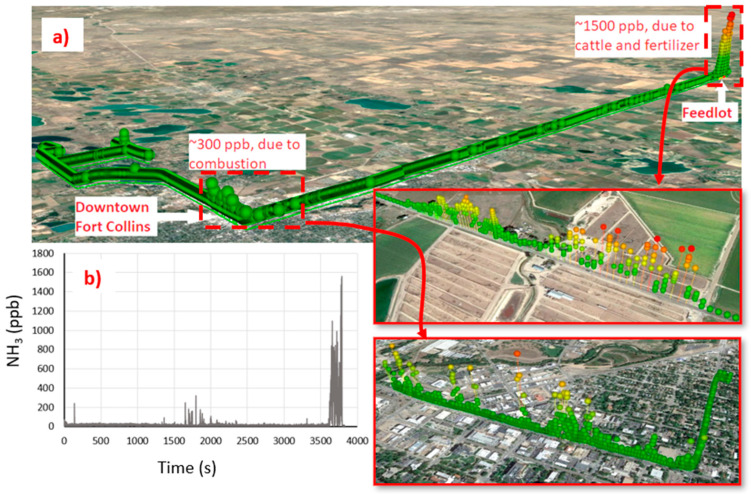
(**a**) Concentration data imported and viewed in Google Earth. (**b**) Ammonia concentration time series from the same data (~1 h measurement).

The preliminary outdoor tests were conducted via vehicle (truck) deployment at ground level using the sensor and vehicle mounting described above. We found this configuration to allow successful data recording including for sensor operation in the presence of vibrations associated with driving on a rugged road with vehicle movement. Figure 7 shows examples of geo-located ammonia readings in the Fort Collins region including in the vicinity of agricultural farms, urban areas and feedlots recorded over approximately 1-h. The data immediately show the utility of capturing elevated ammonia concentrations from a mix of sources including automobile combustion (in the city sensor) as well as from cattle and agricultural sources (feedlot).

#### 3.2.2. UAV-based Measurements

**Figure 8:**
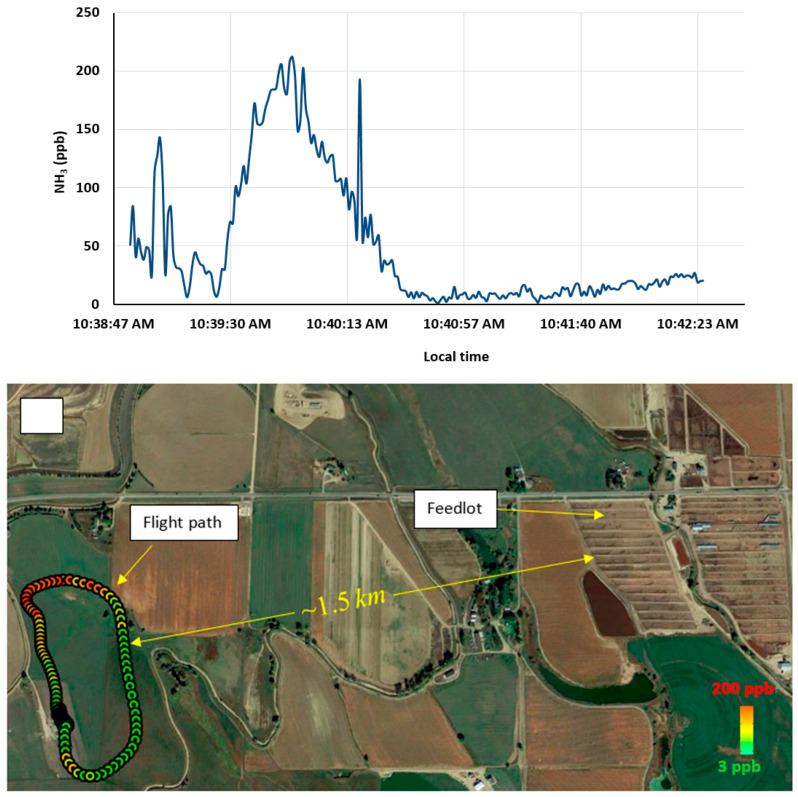
(**Top**) The aerial view of the flight path and the feedlot. (**Bottom**) The measured ammonia concentration timeseries.

We have also shown the ability to deploy the sensor on a UAV to record aerial ammonia data. Again, the sensor and mounting are as described above, though in this case with the UAV mounting configuration. The aircraft used was a fixed-wing Senior Telemaster piloted by a team of CSU flight personnel. The runway used was hardpacked ground on farmland, which illustrates the versatility of the approach (drone style UAV would have even easier logistical needs). The flights were performed at a site which was ~1.5 km away from a big feedlot. Flight times for the Telemaster are relatively short (depending on payload and flight-speed) with the flight shown in Figure 8 being of duration ~3 min. As can be seen from the figure, the ammonia concentration is recorded along the timeseries of the flight path, with altitude and latitude/longitude also being logged from GPS by the sensor. The ammonia concentration data reveal elevated readings up to ~200 ppb associated with the plume (emission) from the feedlot as was also confirmed from the local wind direction at the time.

## 4. Conclusions and Future Work

We have presented the design and performance of a sensitive and compact ammonia sensor based on WMS using a QCL source operating at the favorable spectral region of 10.3 μm. There is a rather complex trade-space among possible ammonia sensors as was summarized in the introduction. In comparison to our past work on CRDS detection in the similar spectral region (and with the same laser), the WMS sensor has slightly poorer detection limit (~2.5 ppb in 3 s for WMS as compared to ~0.6 ppb for CRDS also in 3 s); the WMS package is far smaller and less power-hungry (~3.5 kg and 30 W for WMS as compared to >10 kg and ~600 W for CRDS). The smaller mass and power-draw are very enabling for practical applications, particularly mobile deployment, whereas the CRDS system was too bulky and power-hungry for most realistic UAV deployments. We have presented laboratory testing of the NH_3_ WMS sensor for sensitivity and accuracy. We have also presented results of initial mobile deployments using both a truck and UAV including detection of multiple outdoor plumes from a range of source types. Future work will include more extensive deployments in a range of ambient conditions to further characterize (and optimize) sensor usage in real, outdoor conditions.

Mobile, particularly aerial, deployment of the ammonia sensor can open the door to multiple measurement possibilities. One example is to make multiple passes (transects) of an emitting source and to use the concentration data, along with wind data, to infer the location and mass-flux of the source. Various algorithms are being developed for such purposes including via Bayesian analysis [39] (assuming a point-source in this case). The measurements can also serve to address our regional problems of ammonia transport and deposition as was discussed in the introduction. One approach here is to pair airborne ammonia readings with airborne methane readings (e.g., [40]) and then to use the latter as a conservative tracer to normalize effects of plume dispersion. With such an approach, the changing (declining) ratio of NH_3_:CH_4_ readings can be interpreted as being due to loss to the ground from which deposition velocity and related parameters of ammonia transport can be inferred [41]. Ultimately, sensitive species-specific mobile concentration measurements can be enabling for characterizing and managing many problems in the air quality arena.

## Data Availability

Not applicable.
